# Harnessing Seed Endophytic Microbiomes: A Hidden Treasure for Enhancing Sustainable Agriculture

**DOI:** 10.3390/plants14152421

**Published:** 2025-08-04

**Authors:** Ayomide Emmanuel Fadiji, Adedayo Ayodeji Lanrewaju, Iyabo Olunike Omomowo, Fannie Isela Parra-Cota, Sergio de los Santos-Villalobos

**Affiliations:** 1 Hawkesbury Institute for the Environment, Western Sydney University, Penrith, NSW 2753, Australia; 2 Food Security and Safety Focus Area, Faculty of Natural and Agricultural Sciences, North-West University, Mmabatho 2790, South Africa; 3 Department of Biotechnology and Food Science, Faculty of Applied Science, Durban University of Technology, Durban 4000, South Africa; 4 Department of Pure and Applied Biology, Ladoke Akintola University of Technology, Ogbomoso 210214, Nigeria; 5 Campo Experimental Norman E. Bouleaug, Ciudad Obregon 85000, Sonora, Mexico; 6 Instituto Tecnológico de Sonora, 5 de Febrero 818 sur, Ciudad Obregon 85000, Sonora, Mexico; sergio.delossantos@itson.edu.mx

**Keywords:** seed endophytes, plant growth and health, plant–microbe interactions, stress amelioration

## Abstract

Microbes perform diverse and vital functions in animals, plants, and humans, and among them, plant-associated microbiomes, especially endophytes, have attracted growing scientific interest in recent years. Numerous plant species thriving in diverse environments have been shown to host endophytic microbes. While endophytic bacteria commonly colonize plant tissues such as stems, roots, and leaves, seed-associated endophytes generally exhibit lower diversity compared to those in other plant compartments. Nevertheless, seed-borne microbes are of particular importance, as they represent the initial microbial inoculum that influences a plant’s critical early developmental stages. The seed endophytic microbiome is of particular interest due to its potential for vertical transmission and its capacity to produce a broad array of phytohormones, enzymes, antimicrobial compounds, and other secondary metabolites. Collectively, these functions contribute to enhanced plant biomass and yield, especially under abiotic and biotic stress conditions. Despite their multifaceted roles, seed microbiomes remain underexplored in plant ecology, and their potential benefits are not yet fully understood. This review highlights recent advances in our understanding of the diversity, community composition, mechanisms of action, and agricultural significance of seed endophytic microbes. Furthermore, it synthesizes current insights into how seed endophytes promote plant health and productivity and proposes future research directions to fully harness their potential in sustainable agriculture.

## 1. Introduction

Endophytes are described as those organisms, such as bacteria, fungi, and archaea, inhabiting the tissues of the plants [1,2]. These plant-associated microorganisms are crucial for seed germination and plant growth. According to Fadiji and Babalola [2], microbial interactions are essential for maintaining plant health and increasing agricultural productivity. Nevertheless, the importance of seed-borne microbiomes, especially seed endophytes, has been underrated. Specifically, plants contain bacterial endophytes inhabiting the seeds at very early developmental stages, which is beneficial for increased crop output and plant health. Additionally, bacterial endophytes with advantageous traits are chosen from diverse plants, integrated to seeds by various infection channels, and then passed on to new plants to help succeeding generations [3,4].

Spermatophyte seeds represent an exceptional stage in the life cycle of plants characterized by their ability to remain dormant for years until a favorable environment triggers their germination [5,6]. The seed microbiome plays a crucial role in seed conservation and in maintaining ideal circumstances for germination and plant growth [7,8]. According to Truyens et al. [3], most of the microbial species inhabiting seeds are often present in the soil; thus, the function of seeds as reservoirs of endophytes has since become arguable. Although endophytic microbes were microscopically detected in the seeds of many plant species [9], the importance of seeds as a source of endophytic microbes has been increasingly questioned [10]. Although Mundt and Hinkle [11] had reported earlier that endophytes were isolated only from damaged seeds, thereby raising doubts about their presence in viable seeds, more recent research has led to a shift in perspective. However, the possible role of the seed microbiome in the plant life cycle has been increasingly recognized, suggesting that seeds may be more actively involved in harboring beneficial microbes than previously believed [12]. The majority of the seed microbial endophytes are found in the protected areas of the seed coat, where they can penetrate the seeds through tiny holes. Additionally, certain seeds’ deeper grooves may have incredibly low population densities of endophytes. When seeds are put on water agar for approximately 48 h, endophytic populations may rise from undetectable levels in the seeds of plants to detectable levels in the radicles of surface-disinfested seeds. According to Adams [13], these bacterial endophyte populations were either undetectable or in an uncultivable physiological state. However, bacterial populations that are thriving outside of plant hosts can also colonize those hosts, leading to structural changes in the bacterial community during seedling emergence and subsequent growth [10,14]. Seed-associated bacteria aid in seed conservation and germination by creating a favorable environment in their surroundings through the synthesis of various metabolites [7,15]. In the early stages of the germination of a seed, seeds take in water and release substances known as exudates that can entice bacteria colonizing the spermosphere, rhizosphere, and even the seedling, giving them the ability to directly or indirectly increase plant growth and vigor [5,16]. However, endophytic bacterial vertical transmission has also been noted [17,18]. Microbiologists are interested in transmitting pre-isolated beneficial microbial strains from one generation of plants to the other via seeds; hence, studies are being conducted with an emphasis on vertical transmission to the offspring of plants. For instance, by altering the microbiome of seed embryos of crops, Mitter et al. [19] have developed a unique method for generating features that are mediated by the microbiome of the seed. Furthermore, 29 bacterial and 34 fungal members have been identified to be transmitted in rice, demonstrating a consistent transmission of microbial taxa from parent to progeny seeds during seed maturation. These vertically transmitted microbes are predominant within the seed microbiome, indicating their possible function in improving plant health and growth [20].

Furthermore, since these microbes live inside the seed, seed quality should be the primary factor taken into account when examining seed endophytes. The combination of genetic potential, germination, physical purity, and the absence of seed-borne pathogens defines seed quality. Interestingly, many plants rely on their seeds to pass mutualistic endophytes from one generation to the next [21]. They improve the capacity of the host to withstand various stressors, including drought, pathogens and insect pests, thereby improving plant fitness and increasing its capacity for competition [21,22].

However, the dynamics, diversity, and ecology of seed-associated microbiota represent the pinnacle of intricate associations between microbes throughout their host plant’s whole life cycle. Innovative research opportunities around plant–microbe relationships are being made possible by the diversity of microorganisms found in seeds and their dynamics. The seed and root symbionts, including bacteria and fungi, play a significant role in determining species-specific plant–soil feedback [23]. Even though studies of plant microbial communities frequently underrepresent the seed microbiome, renewed interest in this topic is generating fresh knowledge about its diversity, dynamics, interactions with the plant and soil microbiome, and interactions with the microbes that are linked to dispersers and pollinators. Our understanding of their ecology, colonization methods, origins, and the impact of the seed endophytic microbiome on plant health, productivity, and development is still in its infancy [12]. In this review, recent studies on seed endophytic microbiomes, including archaea, fungi, and bacteria were explored while the diversity, mode of colonization, and transmission of seed endophytic microbiomes were discussed. Similarly, the applications of microbial seed endophytes in the improvement of plant growth, antagonism to phytopathogens, and stress amelioration were examined. To conclude, the connectivity between seed endophytes and seed quality were highlighted with a focus on future research needs that can improve sustainable agriculture.

## 2. Why the Attention on Seed Endophytic Microbiomes?

Endophytes are microbes that live inside the organs of plants, and they typically go unnoticed [2,24], because they rely on their hosts for nourishment and, as a result, have a significant influence on the physiological metabolism of the host by enabling them to withstand various challenges [25]. The endophytes and their respective hosts develop a significant interaction of coevolution and beneficial symbiosis throughout this prolonged association. Endophytes’ genetic make-up and metabolic processes improve and complement their hosts’ metabolic pathways and associated gene expression [26]. A variety of endophytes are transported by seeds. The first variety of microbes to colonize plant tissues is known as seed endophytes, and they form the foundation of the plant’s endophyte population, which may be vertically transmitted to new plants. According to earlier research, specifically, the primary mechanism of progeny infection is vertical transmission of seed endophytes [27]. Three major identified transmission strategies include (i) via the vascular xylem or bundle of host plants; (ii) direct transmission to the endoderm from gametes in pollen; and (iii) direct transmission from matured fruits. In the process of germination, endophytes from the parent plant are passed on to all seedling progeny [28].

Seeds carry out notable functions in the life cycle of spermatophytes, which can remain dormant and inactive for a long period before sprouting into a new plant when the conditions are right [29]. Seed-borne microbial endophytes, often believed to support seed conservation and aid germination of the seeds in soil [7,30], are likely beneficial to seeds. Because they are transmitted vertically across consecutive plant generations, seed-borne endophytes are especially significant because this ensures their existence in the next generation of seedlings [31]. In a bid to assist plant development and growth, this vertical transmission process suppresses microbial pathogens. This mutualism promotes and supports microbial growth as well as plant survival [2]. Thus, fungal and bacterial endophytes found in seeds not only play a critical role in plant development and defense but also benefit the host plants by passing along beneficial endosymbionts to their progeny [31,32].

## 3. Diversity and Community Assemblage of Seed Endophytic Microbiome

According to Hardoim et al. [33], the internal microbial community of plants is made up of complex endophytic bacteria, archaea, and fungi. The seed microbiome, on the other hand, only includes a small variety of microbes [3]. These organisms appear to have developed through co-selection with the host plant species and offer unique growth features for the survival of plants [34]. The endophytic microorganisms that live inside seeds are transmitted from previous generations through the seed; thus, being able to withstand desiccation and harsh climatic conditions [3]. However, with the aid of cutting-edge sequencing technology and microscopy, the seed microbiome may be more thoroughly evaluated to increase the collection of seed-associated microbiomes [12].

### 3.1. Bacteria

Non-culturable and Culturable bacterial endophytes have been identified successfully from several internal plant tissues without harming the plants [10,35]. Notably, rhizospheric and endophytic microorganisms are actively involved in plant growth promotion [36]. In comparison to rhizospheric and pathogenic bacteria, bacterial endophytes are present in plants at lower concentrations [37,38]. Due to reduced competition from other microbes in the surrounding environment for food and host space, as well as the fact that endophytic microbes are already well adapted to inhabit the tissue of plants, seed endophytic microbes benefit from direct infection of plants across generations [3,39]. Nevertheless, the structure of the microbial community is altered during the establishment of seedlings when bacteria from the surrounding environment colonize the host plant [40,41].

Similarly to how bacterial endophytes were found on the inside of seeds [10], they were more frequently found in damaged seeds [11,42]. Most of the safe zones next to the seed coat were also home to seed endophytic bacteria. It was suggested that they may have entered such locations through tiny gaps close to the seed coat [14]. Endophytic microbes are introduced by seed treatments and colonize the tissues of freshly emerging seedlings’ radicles, demonstrating the significance of the spermosphere as a source of these microorganisms [40,43]. Therefore, it appears that the colonization of endophytic microbes begins with the passage of endophytes from the germination slit to the endosperm, from which these endophytic microbes reside in the coleoptile and radicle, resulting in their spread inside the plant’s tissue [44], eventually reaching the seeds, and finally passing from one generation to the next [45,46]. Bacteria are chosen for the seed maturation process depending on their phenotypic characteristics and diversity. Gram-negative bacteria were more prevalent during the early phases of the development of seeds, whereas Gram-positive bacteria increased in number as the seeds matured, according to Mano et al. [47]. *Methylobacterium* sp. and *Sphingomonas* sp. were more prevalent in the early stages, whereas *Bacillus* sp. and *Curtobacterium* sp. were observed to predominate in the latter stages [47]. There are 4 phyla connected to 131 bacterial genera and 25 plant species as seed endophytes in nature [39]. The majority of seed-borne endophytes are composed of proteobacteria, which have 80 genera and mostly 41 genera. The most significant groups include Firmicutes, which have 20 genera, and Actinobacteria, which have 25 genera. Only 6 genera can be found in the phylum Bacteroidetes. Specifically, a study on rice seeds indicated that Gram-negative isolates predominated throughout the earliest stages of seed formation, with Gram-positive isolates emerging more often as the seeds developed [48].

An important indicator of the makeup of all plant endophytic phyla is the phylum of bacteria found in seeds [3]. Actinobacteria, Firmicutes, and unquestionably Bacteroidetes are only infrequently characterized, but Proteobacteria are generally regarded as leading endophytic phyla derived from a variety of plant species. It is particularly difficult to cultivate seed endophytes because of their unique environment, however, the advent of next-generation sequencing has helped with the identification of non-culturable endophytes [49]. *Bacillus* and *Pseudomonas* are two bacterial genera that are frequently discovered in the seeds of different host plant species [50]. Additionally, the majority of the bacteria are discovered inside the seeds, including *Acinetobacter*, *Micrococcus*, *Pantoea*, *Staphylococcus*, and *Paenibacillus* [3]. Proteobacteria, notably class Gammaproteobacteria, dominated the cultivable seed endophytes in the endosphere of rice [51]. *Cistanche phelypaea* seeds were dominated by strains of bacteria from the phyla Actinobacteriota and Proteobacteria [52].

### 3.2. Fungi

**Figure 1:**
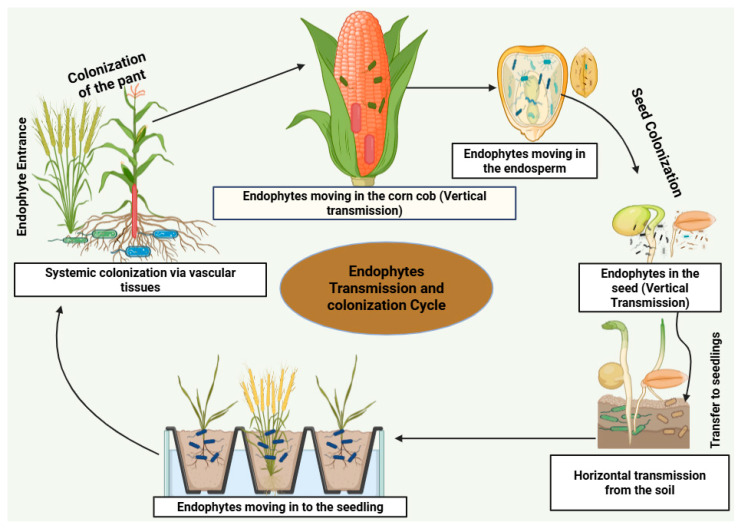
Overview of vertical and horizontal transmission of seed-borne endophytes.

The most varied and often seen microorganisms in many plants are endophytic fungi. According to Faeth and Fagan [53], all plants were designed to be the hosts of endophytic fungi. A layer of tightly interwoven hyphae, indicative of endophytic fungal colonization, was identified within the nucellar remnants located between the seed coat and the aleurone layer of the darnel seed [14,54]. The whole synchronized life cycle of the relationship between plants and endophytes was originally elucidated by Freeman [54], who also clarified how fungal hyphae entered the embryo well before seed development. Additionally, asexual fungi have a remarkable capacity for vertical transmission. By vertically transmitting hyphae through the seeds, which later transform into a mutual diaspore for symbiont and plant, mutualistic plant–fungi associations are demonstrated [55]. The local environment, not the genotype of the host, has a significant influence on the assortment of fungal seed microbiota [56]. Nevertheless, in the majority of cases, the precise location of endophytic fungi in host seeds is not well understood; they may primarily reside in or on the seed coat [57], from whence they may be passed on to subsequent generations either vertically or horizontally [12]. Many Epichloae are classified as “truly seed transmitted” because, in contrast to seed-borne microorganisms that have more passive connections with the host partner, they are completely reliant on the success of plant reproductive stages for their survival and dispersal [2,58] (Figure 1).

The most prevalent seed endophytic fungi are members of the Epichloae genus and its Neotyphodium asexual forms, which are widely recognized for their close ties to the *Poaceae* family. These fungal endophytes that are connected with seeds help plants remain healthy by shielding them from diseases and offering a variety of additional advantages [59]. Yeast, ascomycete, and basidiomycete species are some other fungal endophytes that are linked with seeds [60]. According to Barret et al. [61], the ascomycete classes Eurotiomycetes, Sordariomycetes, Dothidomycetes, and Leotiomycetes, as well as the basidiomycete class Tremellomycetes, are dominated by seeds from the Brassicaceae family. The dominant class of filamentous ascomycetes is the Dothideomycetes, which includes various genera such as *Alternaria*, *Pyrenophora*, *Aureobasidium*, *Cladosporium*, *Stagonospora*, *Phoma*, and *Phaeosphaeria*. The often encountered endophytic genera *Stemphylium*, *Chaetomium* (and associated teliomorphs), *Fusarium*, *Microdochium*, and *Xylaria* are also included in the other classifications of ascomycetes [61]. Although certain fungi particularly live within seeds, the majority of fungal species are associated with the soil from whence they enter the plants by horizontal transmission [12].

### 3.3. Archaea

The world of endophytic archaea is still mostly unknown, but researchers have proposed positive interactions with plants as archaea are ubiquitously found with healthy plants, and their structural distinctions from bacteria contribute to functional changes in the microbiome inhabiting the plant [62]. These symbiotic relationships enhance the capacity of the host plants and archaea to thrive in harsh conditions [63]. Previous studies have shown that archaea are associated with *Candidatus Altiarchaeum* and *Thiothrix* sp. and that they may have a role in the formation of biofilms [64] and in the anaerobic oxidation of methane, which results in CO_2_, a less powerful greenhouse gas [65]. Archaea have not been linked with any human diseases yet, in contrast to fungi and bacteria [66]. However, this might be attributed to a dearth of information on the archaeal ecology and roles of archaea in various systems. Although archaea constitute a significant part of microbial communities associated with the plant and may be important for agricultural uses, little is known about their effects [67]. Only about 1% of archaea in the natural environment have so far been described because many cannot be grown using standard techniques due to unknown growth conditions and nutrient needs [68]. Similarly, it was discovered that just 2% of the total 16S rRNA gene sequences in a study of 146 soils were of archaea when utilizing a set of universal primers for archaeal and bacterial taxa [69]. Novel endophytic archaea such as those belonging to the phyla *Euryarchaeota*, *Thaumarchaeota*, and *Crenarchaeota* were more abundant in the organic than the inorganic fertilizer regime. Newly reported maize endophytes include the genera *Methanococcoides*, *Halomicrobium*, *Cenarchaeum*, *Pyrococcus*, *Methanosarcina*, *Natrialba*, *Nitrosopumilus*, *Methanosphaerula*, *Methanococcus* and *Staphylothermus* [70,71], while *Crenarchaeota*, *Thermoplasmata* and *Methanococci* were the most prevalent archaeal groups. Shi et al. [72] used T-RFLP data with principal component analysis and cluster analysis to demonstrate that archaeal endophyte diversity in sugar beetroot peaks during tuber formation and falls to its lowest during sucrose accumulation. However, numerous additional research projects [52,73] to identify seed-borne endophytic archaea present in rice and common beans were successful. Because there are currently few investigations on seed endophytic archaea, future research should explore this microbiome’s significance and potential applications.

### 3.4. Endophyte–Pathogen Interactions

Endophytic communities encompassing bacteria and fungi coexist alongside a variety of different microorganisms, including other fungi, archaea, non-pathogenic viruses, and bacteria. Bacteria in the rhizosphere have been recognized as vigorous competitors of harmful fungi [74]. Recent studies have revealed that bacterial endophytes have the potential to improve plant growth by reducing the ability of *Clavibacter michiganensis* subsp. michiganensis to systemically infect tomato seeds through tomato fruit lesions and xylem while being protected by intra- and intercellular populations [75]. The bacteria actively grow and move in the fruit’s mesocarp and nearby xylem vessels as the fruits begin to ripen, thereby facilitating the entry of *C. michiganensis* into seeds and fruits, leading to subsequent infections [76]. Plant infections that spread through seeds exhibit the parasitic traits of adaptation by penetrating seeds, surviving seed treatments, invading sprouting seedlings, and ultimately reaching universal dissemination. The accidental cause of tomato canker and wilt, *C. michiganesis*, causes epidemics over the whole region; hence, keeping a healthy seed supply of tomatoes is of utmost importance [77]. The systemic motility of phytopathogens to the seeds was the subject of the earliest research on the bacterial canker disease, but many histopathologic investigations were unable to clearly define this natural method for the contamination of seeds [78]. However, only a small number of bacterial cells were implicated in seed colonization, just beyond the endosperm and xylem vessel attachment sites [76].

## 4. Mode of Transmission of Seed-Borne Endophytic Microbiomes

Depending on the environment, the diverse metabolic properties of fungal and bacterial endophytes found in seeds are employed to aid the host plant’s development. This increases the advantages provided to the host plant and increases its fitness relative to other plants, which might have an impact on the environment as a whole [79].

### 4.1. Horizontal Transmission

Microorganisms penetrate seeds both vertically and horizontally [80] (Figure 1). The mode of transmission is dependent on the three main pathways of transmission, which are (i) the internal pathway, which starts from the seed or the soil at the time of germination through the non-vascular tissues and xylem; (ii) the floral pathway through the stigma of the mother plant; and (iii) external pathways, where seeds become colonized by microbes in the immediate environment [31].

The internal pathway is the primary contributor to vertical transmission; however, the transfer of microbes through pollen, which follows the floral route, may also involve vertical transmission [81]. These two transmission routes relative significance is still unclear, but both are expected to contribute to the seed microbiome diversity [82]. Given that seeds from plants grown in aseptic conditions have lower microbial diversity than seeds from plants grown in natural conditions [34], the majority of the bacteria associated with seeds, including a wide range of general endophytes, are acquired from the environment of the plant [83]. Given the potential advantages that seed endophytes may offer the plant, plants may develop mutualistic connections over time and vertically pass those associations to their progeny [84]. In contrast to the microorganisms associated with seed coats, which are transferred horizontally, the endosperm and embryo-related seed microbiota are frequently transmitted vertically or directly [61]. Additionally, vertical transmission of microbes occurs via gametes, which colonize the embryo before settling in the endosperm [1].

### 4.2. Vertical Transmission

Seed endophytes must have effective motility to penetrate and settle in the seed tissue through a variety of techniques. They are transferred vertically via transgenerational pathways or through vascular connections between the vegetative plant parts, the seed, and parental plants into the seed endosperm [27]. There are three main ways by which seed-borne microbes can spread (i) non-vascular or xylem tissues in the maternal plant; (ii) floral pathways, through the stigma of maternal plants; and (iii) exogenous pathways, whereby seeds are contaminated from the outside environment [85]. Uncertainty surrounds the relative role of vertical and horizontal microbe transfer to plants [82]; however, vertical transmission often occurs in ubiquitous endophytes [27]. This method of transmission is intriguing because it can strengthen a plant by establishing a favorable endophytic population that can be passed along with its advantageous characteristics to the progeny [86]. An evolved form of benign parasitism or mutualism in connection with the host plant is shown by the conservation of vertically transmitted endophytes [87] (Figure 1). In several plant species, vertical transmission of seed-borne endophytic bacteria and fungal endophytes has been found. For instance, isolates of *Microbacterium* spp. and *Bacillus* spp. recovered from the switch grass seeds were also identified in plants grown from these seeds the following year, illustrating vertical inheritance [88]. Furthermore, wheatgrass seeds and mature plant tissues contained the same endophytic bacteria species, confirming seeds as a significant source for passing on endophytes between generations. In addition, whereas endophytic fungi were first believed to be transferred horizontally, reports of their vertical transmission in a number of plant species have been made [89,90,91].

In contrast to epiphytes, which colonize seed surfaces horizontally, the seed microbiota that accompanies the embryo and endosperm is frequently transferred vertically [92]. Typically, horizontal transmission has been seen in rhizosphere bacteria or seed coat microbes. Endophytes can be introduced into seeds via the chalaza and funiculus within the seed endoderm or by way of the micropyle from vegetative portions of plants [93]. Additionally, they are passed on directly through gametes by colonizing the embryo and subsequently settling in the endosperm [1]. This direct transfer from parents to offspring is referred to as vertical transmission [94], and it is a significant method of transmitting endophytes from one generation to the next [3,91].

Endophytic bacteria spread vertically in a variety of plant types; for instance, similar *Enterobacter* sp. were found in rice seeds across successive generations [9], and similar transmission was reported in tomato seedlings [95]. It was shown that maize plants had a basic microbiota, consisting of the same bacterial species and that this microbiome was maintained across subsequent evolutionary barriers, human selection, and subsequent migration across continents [87], and genetically related maize hybrids share the same endophytic species and taxa [96]. The population of obligate endophytes in *Brassica napus* plant roots was compared between fields that had been contaminated with Pb, Cd and Zn, and a control field that had not been contaminated. An association between the populations of root endophytes in the two fields was demonstrated, indicating that the same endophytic community was present in the plants grown in the two different locations, raising the possibility that those obligate endophytes may have arisen through endophytic fusion [97].

Precise information at the strain-level is required to illustrate the vertical transmission of endophytic bacteria; however, its occurrence is noteworthy because it enables plants with a well-established endophyte population to pass on bacteria with desirable traits to their offspring [86]. Vertically transmitted preserved bacteria point to a sophisticated type of mutualism or benign parasitism with the host plant [87]. For example, the vertical transfer of bacterial endophytes may ensure the persistence of bacterial populations in *Eucalyptus* trees [86]. Similarly to this, the seed endophytes in *A. thaliana* were not picked at random but rather largely based on the bacterial traits and environmental challenges that the maternal plant was experiencing [3]. In contrast, because most seed endophytes are ubiquitous across a wide range of climatic circumstances, they may use seeds for their dissemination [34]. Comparable bacterial communities were found in the endospheres of rice, maize, and soybean, demonstrating conserved endophytic assemblages across crop species [87,98].

## 5. Potentials of Seed Endophytic Microbiome in Sustainable Agriculture

**Figure 2:**
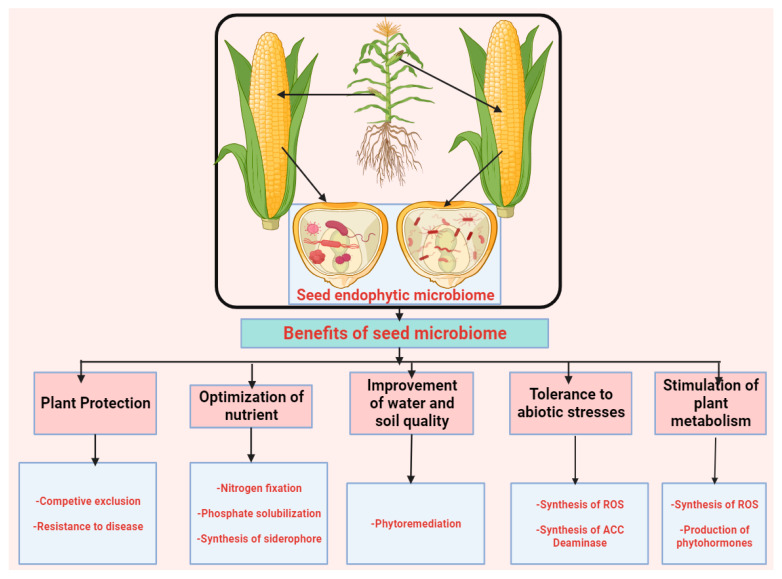
Overview of the beneficial roles of seed microbiomes in plant growth improvement.

There is still limited information on the potential applications and functions of seed-borne endophytes, as most studies focus on their use to boost host plant development, while several studies have also examined their roles as biocontrol agents and in phytoremediation (Figure 2). The uses of seed endophytes are often the same as those seen in other plant components; however, only certain capable endophytes may be present inside plant tissues under unfavorable circumstances [99]. The term “competent endophytes” refers to bacteria that successfully colonize plant sections, can stimulate the physiological system of plants, and are preferred over others, leading to a positive interaction between plants and microbes [33]. Ecology and functions within the host plant are rarely explored for the majority of endophytic microbes. By creating plant hormones and altering their hosts’ ethylene levels, some particular seed endophytic microbes are implicated in influencing the physiological behavior of their hosts [34].

According to Truyens et al. [3], the seed microbiome generally contains a small number of endophytic species, which appear to have been formed by a process of co-selection with the host plant species, benefiting the plant by giving it essential qualities for survival [34]. As a result, both the plant and microbial genomes are used in studying the properties and evolution of plants [100]. The seed microbe genes complement those encoded in plant chromosomes. Because of the distinct plant microbial features that are important to agriculture, plant breeders should promote the best plant–microbe interactions for optimum plant performance. Recent research has shown the effectiveness of a revolutionary strategy known as EndoSeed^TM^, which involves inoculating seeds with helpful microorganisms to increase plant characteristics and related microorganisms in a well-defined manner. At the start of seed production, a helpful microbial strain is introduced into the plant. This theory has been supported by earlier research on the plant growth-promoting *P. phytofirmans*, which, when introduced into the flower, started colonizing the developing embryos by entering the style and stigma, subsequently establishing within the mature seeds [19]. In this manner, the microorganism becomes a component of seeds, beginning to multiply inside the seed before passing on to the following generation in the course of seed germination [101].

Nelson [12] examined the significance of the floral microbiome to the seed microbiome. For instance, Dutta et al. [102] showed that the introduction of non-pathogenic and pathogenic bacteria causes a significant degree of infestation in seeds. Moreover, interior seed tissues may be more immediately accessible to microbes living above the stigma compared to other seed tissues [103]. Microbes have a higher chance of surviving inside the endosperm and embryo than the testa once they have entered the seed [104]. The likelihood of improved seed colonization and subsequent transmission to offspring seedlings improves with the increased number of microbial populations utilized for inoculating the flowers (10^3^–10^9^ cells/bloom) [105].

These annotations suggest that bacteria that are colonizing a flower either externally or internally may be incorporated into the microbiota of seeds and may significantly pass on notable plant traits to the offspring seeds, which has broad applications in agriculture, biotechnology, and related sciences [12,106]. The host plants’ fitness and growth, alongside resistance to various stresses, are all improved by endophytic fungi. According to Jia et al. [107], these modifications play a role in the host plants’ synthesis of metabolites that may be used to manufacture improved medications from pharmaceutical plants.

Phosphate solubilization, N-fixation, increased photosynthetic activity, stimulation of the plant defense system, production of antibiotics, and availability of micronutrients are a few additional factors involved in the enhancement of plant growth [108]. Additionally, biotransformation of heavy metals and organic pollutants biodegradation may be beneficial in enhancing host fitness. If seed or plant endophytes establish synergistic interactions with the host [3,109], these benefits are quickly enhanced. As they compete less for resources and space with microbes inhabiting the external environment and have adapted to the internal plant environment, the microbial endophytes residing inside seeds benefit from quick infection of subsequent generations of plants. Going forward, the potential benefits of seed endophytes are discussed in the subsections below.

### 5.1. Direct Plant Growth Promotion

Bacterial communities associated with seeds can promote the growth of the host plant through diverse metabolic activities influenced by local environmental factors, thereby boosting plant fitness and conferring competitive advantages, which have an impact on how the ecosystem functions as a whole [110]. For example, seed-associated *Bacillus* sp., *Pseudomonas* sp., and *Acinetobacter* sp. promoted the development of carbon cactus by solubilizing the rock minerals, demonstrating the uses of seed endophytes. The weathering of rocks was accelerated by bacterial endophytes isolated from cactus seeds, which were then implicated in soil formation and plant development promotion, assisting in the establishment of cactus seedlings in arid desert regions [111,112].

Johnston-Monje and Raizada [87] reported the evolution of a set of seed endophytes that can survive hundreds of seed generations, suggesting that some endophytes may form long-term associations with their hosts and thus trump the boundaries of ecology, human selection, and evolution. The inoculation of seeds with a variety of bacterial isolates, according to Thomas and Shaik [95], not only improved seedling development and germination but also induced the growth of additional plant endophytes. In unfavorable or harsh climatic circumstances, seed-borne bacterial endophytes can protect and enhance plant development. For instance, when seed-borne *Bacillus* or *Microbacterium* strains that were involved in the synthesis of various hormones, such as indole-3-acetic acid (IAA), volatiles like butanediol and acetoin alongside cytokinins, were administered to switchgrass seedlings, plant development was accelerated. Additionally, the *Bacillus* strain contributed to the solubilization of phosphate [88]. Another seed-borne *Bacillus* strain that may have fixed atmospheric nitrogen (N_2_) and produced ACC deaminase stimulated tomato root and plant shoot development [113].

Under stressful circumstances, seed-borne endophytes from cactus enhanced plant growth augmentation for at least a year without fertilizer addition or any stress signs [111]. These endophytes were later found to be responsible for nitrogen-fixing and producing organic acids that solubilize phosphate in broken rocks, thus creating mineral nutrients [45]. Through potato bioassay, a *Burkholderia* strain engaged in phosphate solubilization, and the synthesis of ACC deaminase was also used to examine the capacity of seed-associated endophytes of maize plants to stimulate plant development. The strain promoted the growth of potato shoots. Additionally, a *Hafnia* strain enhanced the production of acetoin, which further improved potato root growth [87]. Although López-López et al. [114] demonstrated that endophytes extracted from seeds are beneficial for plants in terms of promoting plant growth, their practical field applications remain limited. These endophyte traits that have been conserved may provide information about the community requirements of host plant seeds and their spermosphere [5]. The ability of endophytes as sustainable alternatives to commercial agrochemicals has been explored [2,115]; however, further research is needed to confirm their effectiveness under various agroecological settings. Table 1 provides a summary of notable studies on the plant growth promotion of seed endophytes.

### 5.2. Biocontrol

Higher population densities of endophytes are important as biocontrol agents, but their relevance in ensuring plant benefits is unclear due to several strain-specific characteristics and a variety of possible mechanisms of action, including direct antagonistic effects, induced resistance, and the production of plant growth regulators and hormones. According to Frommel et al. [130], there was a positive correlation between high *Pseudomonas* sp. populations and the potato roots that led to improved plant development and potato yield. *E. cloacae* strain 501R3 reduced the damping-off illness, which in most hosts is caused by *P. ultimum*. According to Roberts et al. [131], this resulted from bacteria competing for the soil’s carbohydrates, amino acids, and lipids in the rhizosphere and spermosphere. In comparison to endophyte-free plants, these host plants have the advantage of being more resistant to a variety of diseases and pests and are more tolerant of harsh environmental conditions [58]. However, endophytes have a wide range of potential for producing different biological products; thus, appropriate culture methods must be developed for commercialization purposes [132]. Additionally, the Orchidaceae endophytic fungi may be grown in submerged and solid media [133], and they can be used to synthesize several vital bio-metabolites for pharmaceutical companies and other industries on a large scale [132].

Numerous seed endophytes also exhibited antifungal properties [134]. For instance, the *Bacillus* and *Microbacterium* isolated from the seeds of switchgrass were responsible for inhibiting the mycelial development of those plants’ fungal pathogens by secretion of specific aflatoxins, such as surfactins, lipopeptide iturin, and mycobacillin [88]. In rice, *Enterobacter* strains producing volatile antifungal compounds, N-acetyl-D-glycosaminidase and ammonia exhibited strong inhibition against *Rhizoctonia solani*, *Heterobasidion annosum*, *Polymyxa myriotyum*, and *Gaeumannomyces graminis* [9]. One-half of the isolates that are inhibitors of *R. solani* and *Pyricularia grisea* were *Bacillus* strains, and some strains of *Xanthomonas*, *Enterobacter*, *Pantoea*, *Cellulomonas*, *Paenibacillus*, *Acinetobacter* and *Stenotrophomonas* with antifungal properties against one or both fungi [135]. Additionally, various fungal species, including *Fusarium oxysporum*, *Pythium ultimum*, and *Curvularia* sp., were inhibited by *Pseudomonas*, *Pantoea*, *Paenibacillus*, *Microbacterium*, and *Curtobacterium* isolated from rice seeds [123]. According to Matsumoto et al. [136], *Sphingomonas melonis* colonizes and is vertically transferred in disease-resistant rice seeds, which gives disease-vulnerable rice plants resistance via producing anthranilic acid.

Seed endophytes are linked to the formation of siderophores and the secretion of various hydrolytic enzymes in addition to their quorum-sensing potentials, which may increase their antifungal activity [137]. By securing the iron, the creation of siderophores improves the competitiveness against pathogens [138]; similarly, the induction of plant gene expression by quorum-sensing molecules boosts the interaction of plants with pathogens and symbionts [139]. *Bacillus* and *Paenibacillus*, which are commercially utilized as biocontrol agents, are linked to the majority of pathogen-reducing endophytes. According to recent observations, the seeds of cucurbits include microbial strains with great potential for reducing the spread of diseases [140]. Therefore, being aware of seed endophytes’ population dynamics is crucial for optimizing their benefits. Depending on environmental variables, including weather or soil nutrient levels, as well as genotypic changes in plants and endophytes [141,142], some endophytes may secrete mycotoxins, particularly alkaloids. Table 2 provides a summary of notable studies on the biocontrol potential of seed endophytes.

### 5.3. Role of Seed Endophytes in Inducing Stress Tolerance

#### 5.3.1. Tolerance to Heavy Metal

Heavy metals (HMs) are inorganic, non-degradable pollutants that are harmful to a variety of living things [155]. They can build up in the body through the food chain and cause neurological disorders, neoplasms, infertility, renal failure, diabetes, cardiovascular disease, and developmental disorders by interfering with cellular activity, causing damage to the DNA, and escalating defects [156,157]. Globally, HMs pollution now poses significant risks to both human health and the environment [158]. Numerous endophytes have been found in the seeds of plants growing in HM-contaminated habitats, and some of these plants exhibited improved resistance to HM. For instance, Mastretta et al. [128] investigated the seed endophytes of tobacco thriving in Zn- and Cd-enriched soils and observed that *Xanthomonadaceae*, *Enterobacter*, *P. fulva*, *Pseudomonas*, *Stenotrophomonas*, *Sanguibacter*, and *C. aminovalericum* had great tolerance to Zn and Cd. Also, Li et al. [159] identified Pb-tolerant *Pseudomonas* from the seeds of tobacco seeds, while Zhou et al. [160] isolated endophytes from rice seeds with high tolerance to Cd, including *Exiguobacterium tabaci* R3-2 and R2-7, *Stenotrophomonas maltophilia* R5-5, *Sphingobium sanguinis* R7-3, and *Pantoea agglomerans* R3-3. Similarly, Cheng et al. [161] reported the isolation of *Sphingomonas* with the Cd tolerance trait from the seeds of rice and Shahzad et al. [162] also identified *B. amyloliquefaciens* with the Cu tolerance capacity in rice seeds. Furthermore, it was discovered that certain HM-resistant strains were recovered from the seeds of other plants in addition to crops. Chu et al. [163] identified 80 seed-borne endophytes from lead–zinc tailings and trash heaps growing *Dysphania ambrosioides* and *Artemisia alpina*. They identified genera including *Cladosporium*, *Alternaria*, *Phoma*, *Plectosphaerella*, *Colletotrichum*, *Ctenophora*, *Peyronellaea*, *Ramularia*, and *Epicoccum nigrum*, where *E. nigrum* exhibited the highest tolerance to Cd. According to Truyens et al. [164], improved host resistance to Cd was linked to the endophytes from the seeds of *Arabidopsis thaliana*. The identified genera include *Mycobacterium*, *Cellulomonas*, *Nocardioides*, *Pseudonocardia*, *Aeromicrobium*, *Flavobacterium*, *Pseudolabrys* and *Devosia*. In addition, the fungal endophyte *Epichloë* from grass seeds conferred resistance to HM on the host [165]. Table 3 provides a summary of notable seed endophytes that are tolerant to heavy metals.

#### 5.3.2. Tolerance to Drought

The physiology of leaf structure, plant roots, nutrient intake, seedling germination, and photosynthetic activity is all significantly impacted by drought, which generally reduces plant development [176,177]. Previous investigations showed that the tolerance of host plants to drought stress can be improved by seed endophytes. The xerophyte’s seeds are made up of many endophytes, some of which have shown improved tolerance to drought. Wang et al. [178] examined the upland rice seeds, and they discovered a variety of endophytes, including *Buttiauxella* and *Curtobacterium*, which had higher drought tolerance than *Pseudomonas*, *Pantoea*, *Methylobacterium*, *Microbacterium*, and *Sphingomonas*. Similarly to this, Jeong et al. [172] reported that the seeds of the invasive xerophyte *Lactuca serriola* also contained a large number of endophytes, including *Exiguobacterium tasmaniensis*, *Kosakonia cowanii*, *Curtobacterium dublinensis* and *Pseudomonas* and *Xanthomonas*, with *K. cowanii* conferring significant resistance to drought. Also, drought-tolerant endophytes have been isolated from wheat seeds [175]. Similarly, Abideen et al. [119] reported that the *Hordeum vulgare* seed endophytes *Pseudomonas* and *Pantoea* can improve the host’s resilience to drought. In addition, from the seeds of *Festuca arundinacea*, [171] also identified the drought-tolerant *E. coenophiala*.

#### 5.3.3. Tolerance to Salt Stress

Salt stress has a significant impact on a plant’s metabolic and physiological functions by reducing photosynthetic activity, seedling development, ion toxicity, and water stress, alongside the rate of lipid metabolism and protein synthesis [179,180]. Seeds of salt-tolerant plants contain numerous endophytes, some of which exhibit greater salt resistance. About 172 bacterial endophytes were identified from mangrove propagules [117], some of which include the *Bacillus*, *Actinobacteria*, *Dyadobacter*, *Corynebacterium*, *Gordonia*, *Micrococcus*, *Enterococcus*, *Nocardioides*, *Staphylococcus* and *Rhizobium*. Specifically, *Glaciecola terrae* KMP456-M40 increased the biomass of rice under salt stress by 62% and the root length of mangrove seedlings by 65%. Similar findings were made by Walitang et al. [181], who showed that all the cultivars of the indica subspecies have similar ribotypes, which may reflect the core microbiome of *Flavobacterium*, *Curtobacterium*, *Xanthomonas*, *Herbaspirillum*, *Stenotrophomonas*, *Microbacterium*, and *Enterobacter*. However, when exposed to salt stress, the endophytic communities of salt-tolerant and salt-sensitive rice cultivars give way to bacterial communities that belong to the genera *Pantoea*, *Flavobacterium*, *Enterobacter*, *Curtobacterium*, *Kosakonia*, and *Microbacterium*. These community shifts suggest that the hosts may be able to withstand salt stress as a result of changed core microbiota.

## 6. Do Seed Endophytic Microbiomes Have Any Connection with the Quality of Plant Seeds?

A positive correlation has been observed between seed quality and endophytic content. High-quality seeds, for example, are typically considered to be disease-free, exhibiting greater vigor and higher germination rates [182]. It has been proven that tall fescue seedlings with seeds that were harvested before physiological maturity had lower endophytic fungus infection rates. Additionally, seeds that are removed before they are fully developed have a lower germination rate and fewer vigorous seedlings [21]. In addition, particular circumstances related to seed storage, such as high temperature and high humidity, might limit the viability of endophytic fungi in tall fescue seeds [183]. Additionally, during seed storage, the number of endophytic bacteria that can be isolated decreases over time [128]. According to Truyens et al. [39] the sterilization procedure used on the seeds before planting had a significant impact on the number of seed bacterial endophytes in *A. thaliana*. Additionally, the endophytic bacteria that are present in seeds may be important for plant development, and when seed bacteria die, germination is inhibited [184]. Inferring from this, it can be said that plants are no longer thought of as monogenetic beings but rather as polygenetic objects and that the plant-associated microbiome plays a crucial part in improving the diversification, fitness, and adaptation of the holobiont [185,186]. Overall, using a variety of biotechnological techniques, endophytes linked with seeds can enhance the quality of seeds, which in turn promotes the establishment of important agricultural products [159].

## 7. Recent Advances in Seed Microbiome Identification and Their Role in Sustainable Agriculture

Identification of critical seed endophytic microbiomes for future applications necessitates a multifaceted procedure that incorporates both traditional and modern methods from molecular biology, bioinformatics, and microbiology. Core taxa, such as *Pantoea*, *Pseudomonas*, *Alternaria*, and *Cladosporium* sp, have been consistently found in various cultivars, geographical locations and plant species [187,188]. Multi-omics methods that include genomics, metagenomics, metabolomics, and metatranscriptomics have begun to significantly improve our knowledge of seed endophytic microbiomes, including the community composition, colonization, interactions with plants, and their potential functions in plant health and growth improvement [189]. On the other hand, bacterial genera such as Candidatus Carsonella, Candidatus Phytoplasma, TM7 group (now Candidatus Saccharibacteria), Wolbachia, and members of Acidobacteria like Koribacter, which are rarely isolated through conventional culturing [190]. Similarly, fungal endophytes such as Candidatus Glomeribacter, Mycoavidus, Bifiguratus, and various unclassified Basidiomycota and Mucoromycotina are frequently detected in seeds via ITS sequencing but remain unculturable [70,191,192]. Furthermore, plant growth-promoting features of a specific synthetic microbial community or a particular microbial strain, such as biocontrol efficacy against phytopathogens and nutrient cycling, may be tested using in vivo and in vitro experiments [193]. Similarly, whole-genome sequencing (WGS) may be used to predict the genetic activities of a microbe [194]. In this sense, most seed endophytes appear to be culturable, facilitating the investigation of their roles and ideal growing conditions. Therefore, assessing the efficacy of microbiological tools on plant production and development is also important, but it is difficult owing to the complexities of plants, climatic variables, and soil [195]. According to most research, utilizing beneficial seed endophytic microbiomes to improve plant yield and production poses negligible environmental hazards and effects on non-target species. Nonetheless, before deploying large-scale uses of microbe-treated seed products, potential ecological consequences must be assessed [196].

## 8. Future Prospects

Plant–microbe interactions play a pivotal role in plant health, productivity, and fitness, driving innovations in agricultural management [180]. Further study on the potential for flower-associated microorganisms to structure seed microbiomes appears to open new avenues for agricultural innovation. Aside from that, knowledge of specific microbial strains linked to particular crop species can significantly help to comprehensively exploit the novel approach of seed endophytic microbiomes in agriculture, not only for preserving productivity and plant health but also for improved germination of seeds [197]. As a result, further study on specific crops is still needed to examine how certain strains survive in inoculated or original seeds and then spread to subsequent generations, with an emphasis on the behavior of the strain in plant offspring. Additionally, selecting seed-associated microbes that confer resistance to plant diseases may contribute to the elimination of specific pathogens—not only within the current host plant but also in subsequent generations—thereby presenting a novel strategy for plant disease prevention. Given that seed endophytes rely entirely on the host plant for their reproduction and survival, their selection should favor mutualistic traits while excluding pathogenic potential. Future research on the seed microbiome that can improve sustainable agriculture offers exciting opportunities by examining the unique traits of seed microbiomee and their chemistry with the host plant, particularly by examining the tolerance of germinated seeds inhabiting the endosymbionts when exposed to different stresses. To fully understand the endophytic mode of action, their genetic transfer into the host, and their use for managing diseases, further research employing advanced molecular tools is essential.

A significant portion of the endophytic population within seeds remains unexplored. Multi-omics approaches will provide deeper insights into the phenotypic traits, dominant genera, and functional roles of seed endophytes in plant development and germination.

Future studies should concentrate on developing endophyte resources and the integration of other novel technologies, such as molecular biology, transcriptomics, and the microbiome, to create solutions that are tailored to particular crops and local conditions, especially in regions with harsh environmental conditions. This can help in the reclamation and development of cultivated land resources, alongside assisting the crops in yield improvement and adaptation to their environment. Furthermore, more studies are needed to better understand seed–endophyte interactions and how they contribute to the molecular induction of defense mechanisms against biotic threats, and to pinpoint the genetic factors influencing seed–endophyte dispersal, vertical transmission, and seed colonization. Also, an in-depth investigation is required to establish the changes that take place in seed-associated endophytes that take place throughout seed growth, storage, and germination to guarantee the generation of seeds of the highest quality.

Furthermore, despite the promise of seed endophytes for crop improvement, the ecology of the seed microbiome is poorly understood, and many issues remain unresolved, even though these helpful microorganisms have been used to increase crop productivity. Taken together, further important questions and knowledge gaps that demand attention are as follows: (i) Do seed endophytic microbiomes display distinctive phylogenetic, taxonomic, and functional trends that differ throughout ecological gradients? (ii) to what extent is the level of host-specificity of endophytic microbiome inhabiting plant seeds, and does it change across time and space? (iii) how stable is the core endophytic microbiome in seeds under abiotic and biotic perturbations, and what variables define the core endophytic microbiome of plant seeds? (iv) how does the seed endophytic microbiome influence ecological interactions among co-existing plant species, most importantly in the early stages of host plant establishment and recruitment? (v) is it environmentally wise to engineer microbiomes in seeds for the conservation of species and restoration of habitats? A more comprehensive understanding of the ecological significance and assembly processes of seed microbiomes in shaping plant community dynamics and structure under variable environmental conditions can be achieved through addressing the aforementioned questions using carefully designed and well-replicated studies.

## 9. Conclusions

Seed microbiomes represent a rapidly emerging area of research with significant potential to support low-input and ecologically sound strategies in sustainable agriculture. They hold considerable promise for improving plant health, productivity, and resilience in both food and non-food crops. While extensive research has been conducted on plant-associated microbiomes in general, seed endophytes remain relatively understudied, despite being a crucial component of the plant holobiont and a key determinant of early microbial community assembly and function. Unlike epiphytes, seed endophytes form stable, long-term associations with their host plants and play a pivotal role in the initial colonization and development of the seedling microbiota. Seeds act as natural reservoirs for diverse endophytic microbes, with their ecological importance largely attributed to their capacity for vertical transmission across generations. This mode of inheritance allows the direct transfer of beneficial microbial partners from parent plants to progeny, enhancing early-stage plant performance and resilience to biotic and abiotic stresses. Our study outlined the taxonomic composition, functional roles, and ecological significance of seed-associated microbial communities, highlighting their interactions with host plants and potential applications in enhancing crop productivity and environmental sustainability. Moving forward, a deeper mechanistic understanding of seed microbiome assembly, vertical inheritance, and functional contributions, especially through the use of multi-omics and well-replicated experimental designs, will be essential for translating this knowledge into practical agricultural innovations and conservation strategies.

## Figures and Tables

**Table 1:** Summary of studies on plant growth promotion of seed endophytes.

Endophytic Microbes	Host Plant	Functions	References
*Epichloë coenophiala*	Festuca arundinacea	Activate stress response mechanisms for protection	[116]
*Gordonia terrae* KMP456-M40	Mangrove propagules	Promotes mangrove root growth	[117]
*Kosakonia cowanii*	*Lactuca serriola*	Drought tolerance in invasive lettuce	[118]
*Pseudomonas* sp., *Pantoea* sp.	*Hordeum vulgare* L.	Improved biomass, mineral balance and antioxidant capacity under drought	[119]
*Bacillus amyloliquefaciens*	*Oryza sativa*	Production of Phytohormone	[120]
*Paenibacillus polymyxa*	*Oryza sativa*	Resistant to pathogens and production of glucanase	[121]
*Microbacterium yunnanensis*, *Exiguobacterium soli*, *Micrococcus luteus*, *Leclercia adecarboxylata*, *Staphylococcus epidermidis*, *Pantoea dispersa*	*Oryza sativa*	IAA production and enhancement of plant growth	[122]
*Acinetobacter* sp., *Curtobacterium citreum*, *Microbacterium* sp., *Pantoea ananatis*, *Pseudomonas* sp., *Paenibacillus* sp., *Pantoea agglomerans*, *Pantoea* sp., *Staphylococcus cohnii*, *Microbacterium* sp., *Rathayibacter larrymoorei*, *Sphingomonas* sp., *Curtobacterium* sp.	*Oryza sativa*	Phytohormone and metabolite production, phosphate-solubilizing, antifungal, plant growth promotion	[123]
*Enterobacter asburiae*, *Pseudomonas putida*, *Pantoea dispersa*	*Oryza sativa*	IAA production, antifungal, phosphate-solubilizing and promotion of plant growth	[124]
*Bacillus*, *Nocardioides*, *Acinetobacter*, *Paracoccus*, *Enterococcus*, *Sphingomonas* and *Phyllobacterium*	*Glycine max*	Phytate-solubilizing	[114]
*Bacillus subtilis*	*Lycopersicon esculentum*	Plant growth promotion, phytohormone and production of metabolite	[113]
*Kosakonia*, *Massilia*, *Pantoea*, *Sphingomonas*, *Burkholderia*, *Pseudorhodoferax*, *Caulobacter*, *Bacillus* sp., *Methylobacterium*, *Microbacterium*, *Curtobacterium*, *Chitinophaga and Mucilaginibacter*	*Triticum esculentum*	Plant growth promotion, production of metabolite and phytohormone	[101]
*Klebsiella palustris*, *Bacillus pumilus*, *Microbacterium fujisawaense*, *Pantoea ananatis*, *Microbacterium radiotolerans*	*Oryza sativa*	Enzyme production, osmotic stress tolerance	[125]
*Neotyphodium oenophialum*	*Festuca arundinacea*	Ergovaline and loline alkaloid production and improved protection against herbivores	[126]
*Alternaria* sp., *Penicillium corylophilum* and *Phoma* sp.	*Invasive Phragmites*	Improvement of seedling growth and seed germination	[30]
*Epichloë ceonophiala*	*Salvadora phoenix*	Improved resistance against herbivores and environmental stresses	[127]
*Diaporthe* sp.	*Citrus ledgeriana*	Production of alkaloid	[128]
*Epichloë typhina*	*Dactylis glomerata*	Improvement of photosynthesis and growth of host plant	[129]

**Table 2:** Summary of other studies showing the biocontrol potential of seed endophytes.

Endophytes	Pathogens	Plant Host of the Endophytes	References
*Moesziomyces* spp.	*Alternaria* sp., *Fusarium* sp.	*Oryza sativa*	[143]
Synthetic bacterial community	*Aspergillus flavus*, *Fusarium oxysporum*	*Arachis hypogaea*	[144]
*Bacillus subtilis* BHN1, *Bacillus* stercoris BHR2, Paenibacillus peoriae YHR2-1	*Fusarium oxysporum* (races 1 and 2), *Botrytis cinerea*	*Solanum lycopersicum*	[145]
*Bacillus velezensis* NEAU-CP5	*Ralstonia solanacearum*	*Solanum lycopersicum*	[146]
*Bacillus halotolerans* strain B33	*Fusarium graminearum*, *Alternaria alternata*, *Aspergillus flavus*	Small-grained cereals (wheat, barley, oats)	[147]
*Bacillus amyloliquefaciens* RWL-1	*Fusarium oxysporum*	*Oryza sativa*	[148]
*Bacillus mojavensis* PS17	*Fusarium oxysporum* ZUM2407	*Triticum aestivum*	[149]
*Bacillus velezensis* ZMW8	*Fusarium verticillioides*	*Zea mays*	[150]
*Pseudomonas aeruginosa* BHUJPCS-7	*Fusarium oxysporum*	*Cicer arietinum*	[151]
*Pseudomonas marginalis* B1	*Fusarium culmorum*	*Brassica oleracea*	[152]
*Pantoea dispersa* BB1	*Burkholderia glumae*	*Oryza sativa*	[153]
*Lactococcus* and *Pantoea*	*Oomycete pathogens*	*Cucurbits*	[140]
*Paenibacillus*	*Fusarium graminearum*	*Triticum aestivum*	[154]

**Table 3:** Overview of notable seed endophytes that are tolerant to stresses.

Seed Endophytes	Stress	Host	References
**Heavy Metal Tolerance**			
*Pantoea* and *Bacillus*	Cadmium (Cd)	*Agrostis capillaris*	[166]
*Bacillus amyloliquefaciens*	Copper (Cu)	*Oryza sativa*	[162]
*Epichloë*	Cadmium (Cd)	*Lolium perenne*	[165]
*Pseudomonas*	Lead (Pb)	*Nicotiana tabacum*	[159]
*Methylobacterium*	Cadmium (Cd)	*Carex pumila*	[167]
*Cellulosimicrobium cellulans*	Copper (Cu)	*Sesbania cannabina*	[168]
*Sphingomonas*	Cadmium (Cd)	*Oryza sativa*	[161]
*Rhodococcus* and *Bacillus*	Copper (Cu)	*Agrostis capillaris*	[169]
*Cellulosimicrobium cellulans*	Copper (Cu)	*Sesbania cannabina*	[170]
**Drought Tolerance**			
*Epichloë coenophiala*	Drought	*Festuca arundinacea Schreb*	[171]
*Pantoea and Pseudomonas*	Drought	*Hordeum vulgare*	[119]
*K. cowanii*	Drought	*Lactuca serriola*	[172]
*Epichloë festucae* var. lolii	Drought	*Lolium perenne*	[173]
**Salt Tolerance**			
*Bacillus mojavensis* PS17	Salt	*Triticum aestivum*	[149]
*Gordonia terrae* KMP456-M40	Salt	Mangroves	[117]
*Pantoea agglomerans* Ed-3 and *Bacillus subtilis* Es-1	Salt	*Elymus*	[174]
*Bacillus aryabhattai*, *Bacillus altitudinis*, *Bacillus gladioli*, *Bacillus wiedmannii* and *Pseudomonas aeruginosa*,	Salt	*Triticum aestivum*	[175]
*Xanthomonas*, *Flavobacterium*, and *Microbacterium*,	Salt	*Oryza sativa*	[51]
